# Polyphenol-Enriched Extracts from Leaves of Mediterranean Plants as Natural Inhibitors of Monoamine Oxidase (MAO)-A and MAO-B Enzymes

**DOI:** 10.3390/nu18010022

**Published:** 2025-12-20

**Authors:** Antonio D’Errico, Rosarita Nasso, Mario Ruggiero, Rosario Rullo, Emmanuele De Vendittis, Mariorosario Masullo, Filomena Mazzeo, Rosaria Arcone

**Affiliations:** 1 Department of Medical, Movement and Well-Being Sciences, University of Naples “Parthenope”, Via Medina, 40, 80133 Napoli, Italy; antonio.derrico002@studenti.uniparthenope.it (A.D.); rosaritanasso@gmail.com (R.N.); mario.ruggiero005@studenti.uniparthenope.it (M.R.); mario.masullo@uniparthenope.it (M.M.); 2 Institute for the Animal Production Systems in the Mediterranean Environment, Consiglio Nazionale Delle Ricerche, Piazzale Enrico Fermi 1, 80055 Portici, Italy; rosario.rullo@cnr.it; 3 Department of Molecular Medicine and Medical Biotechnologies, University of Naples Federico II, Via S. Pansini 5, 80131 Napoli, Italy; 4 Department of Economics, Law, Cybersecurity and Sport Sciences, University of Naples “Parthenope”, Via Della Repubblica, 32, 80035 Nola, Italy

**Keywords:** polyphenol extracts, neurodegenerative disorders, Alzheimer’s disease (AD), Parkinson’s disease (PD), monoamine oxidases inhibitors, MAO, Mediterranean plants

## Abstract

**Background**: Alzheimer’s disease and Parkinson’s disease are multifactorial disorders causing severe disability, rising with the increase in life expectancy. Currently, the identification of natural compounds useful against these disorders is becoming an urgent necessity. In this study, we used polyphenol-enriched extracts obtained from leaves of Mediterranean plants, which are important in animal feeding (*Lotus ornithopodioides*, *Hedysarum coronarium*, *Medicago sativa*) and in the human Mediterranean diet (*Cichorium intybus*). **Objectives**: The aims of this study were as follows: (i) tentative identification of the organic compounds present in the extracts; (ii) determination of their effect on the activity of monoamine oxidase (MAO)-A and MAO-B, key enzymes involved in the metabolism of aminergic neurotransmitters, as well as on protein expression level of these enzymes in cell lines expressing basal MAO-A and MAO-B. **Methods**: The ability of plant polyphenol extracts to inhibit MAO-A and MAO-B activity was assessed by in vitro enzyme assays. The protein expression level was analyzed by Western blotting. **Results**: Our data demonstrate that all the extracts behaved as MAO-A and MAO-B inhibitors, although to a different extent and enzyme inhibition mechanism; among them, the extract from *L. ornithopodioides* induced a decrease in MAO-A protein level in human AGS gastric adenocarcinoma and SH-SY5Y neuroblastoma cell lines. **Conclusions**: These data reinforce the hypothesis that a plant-based diet and/or integrative supplementation of pharmacological treatments can be considered for preventing and relieving symptoms of neurodegenerative diseases.

## 1. Introduction

Neurological disorders, encompassing Alzheimer’s disease (AD) and Parkinson’s disease (PD), cause severe disabilities, cognitive impairments, and death [1,2]. In recent years, their incidence has been growing due to the increase in life expectancy [3,4].

The specific pathogenic mechanisms of these neurodegenerative disorders are not fully understood. However, current leading hypotheses propose that AD is characterized by abnormal protein aggregation, specifically β-amyloid (Aβ) plaques and hyperphosphorylated Tau proteins [5], while PD is primarily associated with dopaminergic neurodegeneration [6], with both conditions ultimately resulting in progressive neuronal death.

The progressive neuronal loss and the decrease in synaptic connections, both correlating with the appearance of clinical symptoms and disease progression, cause severe disabilities of movement (rigidity, bradykinesia, slowness, walking difficulties, tremor) as well as aphasia, disorientation, and depression [7].

In these multifactorial disorders, a key role is played by enzymes affecting the metabolism of neurotransmitters, such as monoamine oxidases (MAOs); indeed, these macromolecules catalyze the oxidative deamination of endogenous and exogenous amines and therefore regulate dopamine, serotonin, norepinephrine, and epinephrine levels [8]. In most mammalian tissues, two isoforms, MAO-A and MAO-B, have been detected, which show different tissue-specific expression, substrate specificities, and inhibitor sensitivities [9,10]. Although both isoforms catalyze the oxidation of dopamine, tyramine, and tryptamine, these enzymes play different roles. In fact, MAO-A preferentially acts on the oxidative deamination of dopamine, thus decreasing its intracellular level, whereas MAO-B regulates gamma-aminobutyric acid (GABA) level [10].

Currently, one of the pharmacological approaches for treating neurodegenerative disorders is based on the administration of MAO inhibitors, and up to now, different synthetic drugs have been identified [11,12,13]. MAO-A is selectively inhibited by clorgyline, causing an increase in serotonin (5-HT) and norepinephrine levels in the brain, leading to an antidepressant effect [14]. MAO-B inhibitors, such as selegiline, rasagiline, safinamide, and KDS 2010, are used in PD treatment because they block dopamine catabolism, enhance dopamine signaling, and selectively elevate dopamine levels in the synaptic cleft [3,15]. However, these diseases cannot be reversed by drugs, and side effects are associated with their use [11,16].

In addition, the multifactorial nature of AD and PD may explain the reason for the lack of an effective and novel therapeutic treatment capable of preventing, delaying, and counteracting the progression of these diseases [17].

To overcome this problem, novel research has focused on the identification of plant-derived natural agents, endowed with neuroprotective properties acting with multi-targeting effects [18,19,20,21], thus representing an alternative or integrative approach to synthetic drugs for their low toxicity and high efficacy.

Under this concern, polyphenols extracted from plants have shown several interesting effects, such as the decrease in the incidence of neurodegenerative diseases, because they act on different cell signaling pathways [22,23,24]. Forage plants, for their high diffusion and availability, represent an abundant and low-cost source to produce flavonoids that exert beneficial effects on human health phytochemicals [25]. Extracts derived from forage crops enriched with polyphenols and tannins have shown beneficial effects on human health, due to the presence of bioactive compounds such as polyphenols and tannins that are involved in several metabolic processes [25,26], thus suggesting an interesting anti-cancer potential [27,28,29,30].

Under this frame, we performed previous studies on polyphenol-enriched extracts from leaves of the Fabaceae plants *Lotus ornithopodioides* (*Lo*), known as Southern Bird’s foot trefoil, *Hedysarum coronarium* (*Hc*), also called Sulla, and *Medicago sativa* (*Ms*), known as Alfalfa, or the perennial herb of the Asteraceae, *Cichorium intybus* (*Ci*), the common Chicory, showing their antioxidant properties for the inhibiton of catalase and xanthine oxidase activity [25,31]. In addition, these extracts exerted neuroprotective effects by inhibiting key enzymes involved in AD and PD, such as acetylcholinesterase (AChE) and butyrylcholinesterase (BuChE), and by reducing the amyloidogenesis process [21]. Therefore, these extracts exert neuroprotective effects by acting as multitargeting agents. In addition, their biological activity cannot be referred to a single class of molecules or components, but rather to a possible synergistic interaction among bioactive flavonoids and condensed tannins, as revealed by the analysis of the chemical composition of *Lo*, *Hc*, *Ms*, and *Ci* [21].

Based on the above, the aim of this study was to characterize the effect of extracts from leaves of *Lo*, *Hc*, *Ms*, and *Ci* on MAO-A and MAO-B activity, by in vitro enzyme assays. In addition, we also examined whether these extracts could modulate MAO-A and MAO-B protein expression in human cell lines that express a basal level of MAO-A, such as the AGS gastric adenocarcinoma cells [32] or MAO-B as the SH-SY5Y neuroblastoma cells [33]. These findings could contribute to identifying novel plant-based strategies for managing neurodegenerative disorders. The results of these investigations showed that, among the different extracts tested, those obtained from *M. sativa* and *L. ornithopodioides* may be useful as a possible coadjutant in the integrative supplementation of pharmacological treatments for the prevention and management of neurodegenerative diseases.

## 2. Materials and Methods

### 2.1. Materials

Human MAO-A and MAO-B, kynuramine, and thioflavine T were purchased from Sigma-Aldrich (Milan, Italy). Trifluoroacetic acid (TFA) and solvents for the HPLC separation were from Carlo Erba reagents (Milan, Italy).

### 2.2. Preparation of Polyphenol-Enriched Extracts from Leaves of Mediterranean Plants

The herbaceous plants used in this study were collected in Italy after flowering, as previously described [31]. Briefly, leaves were deep-frozen, powdered, defatted with chloroform, and then extracted. For *L. ornithopodioides* and *H. coronarium*, the process began with a water/acetone extraction, followed by chromatography. For *M. sativa* and *C. intybus*, extraction was performed with 80% methanol overnight. After centrifugation, the supernatant was collected, and the residue underwent a second extraction. The solvents were then removed under vacuum, and the samples were freeze-dried and dissolved in dimethylsulfoxide (DMSO, Merk Serono, Roma, Italy).

### 2.3. HPLC Analysis of Extracts from Leaves of Mediterranean Plants

The origin of four selected Mediterranean plants, including *Lo*, *Hc*, *Ms*, and *Ci*, and the preparation of extracts from leaves of these plants have been recently reported [31]. In particular, the samples were called *Lo*CT, *Hc*CT, *Ms*F, and *Ci*F, because enriched with condensed tannins (CT) or flavonoids (F), respectively. A previous characterization of their organic composition included the total phenolic content, flavonoids, and proanthocyanidins, evaluated as gallic acid, catechin, and delphinidin equivalents, respectively [31]. The concentration of the extracts was thoroughly expressed as gallic acid equivalents; 1 µM of gallic acid corresponded to 0.17 µg/mL.

A focus on the organic composition of the extracts was realized through an HPLC analysis. To this aim, separation of *Lo*CT, *Hc*CT, *Ms*F, and *Ci*F samples was achieved at 25 °C by the Breeze HPLC system purchased from Waters S.p.A. (Sesto San Giovanni, Milan, Italy), using a Synergi Max-RP C12 column (250 mm × 4.6 mm, 4 μm particle size) purchased from Phenomenex (Torrance, CA, USA). Solvents used were as follows: solvent A, milliQ water containing 0.05% TFA and 1% methanol; solvent B, acetonitrile containing 0.05% TFA. The organic components were identified and quantified by comparing the area and peak position in each sample chromatogram (at least triplicates) to the corresponding data obtained from reference compounds. The reference compounds used in the HPLC analysis are reported in the Supplementary Materials.

### 2.4. Monoamine Oxidase Assay

The activity of MAO-A and MAO-B was assayed by a fluorimetric method, as previously reported [20,32] using a Cary Eclipse Spectrofluorimeter (Agilent, Milan, Italy). The method, based on the oxidation of the substrate kynuramine by both MAOs, leads to the production of 8-hydroxychinoline, which becomes fluorescent in alkaline conditions. Indeed, the fluorescence signal was recorded, using an excitation and emission wavelength of 315 and 380 nm, respectively, and setting both excitation and emission slits at 10 nm. A 250 μL reaction mixture was prepared in 50 mM potassium phosphate (Merk Serono, Roma, Italy) buffer, pH 7.1, and contained 40 μM (in the steady state activity measurements) or 25–150 µM (in the kinetic experiments) kynuramine in the absence or in the presence of different concentrations of the various extracts. The reaction started with the addition of 2.5–5.0 µg or 2.1–4.2 µg MAO-A or MAO-B, respectively; after 20 min incubation at room temperature (20–25 °C), the reaction ended with the addition of 150 μL of 2 M NaOH. Then, after an additional 10 min, the mixture was supplemented with 240 μL of water and centrifuged for 10 min at 15,000 rpm. Finally, 500 µL of the supernatant was used for measuring its fluorescence. Blanks run in the absence of enzymes, due to the fluorescence of the extract, were carried out in parallel and subtracted. In the steady state activity measurements, the residual MAO-A or MAO-B activity was compared to that measured in the absence of the extracts and expressed as a percentage. The data were collected in at least three different experiments. Clorgiline and selegiline were used as positive controls of MAO-A and MAO-B inhibition, respectively [11]. The concentration of extracts leading to 50% residual activity (IC_50_) was calculated from semilogarithmic plots in which the logarithm of the ratio of residual activity was plotted against the extract concentration.

The inhibition power and mechanism of inhibition were evaluated through kinetic measurements of the kinetic parameters *K*_m_ and *V*_max_ of MAO-A and MAO-B activity in the absence or in the presence of different extract concentrations, as previously reported [31].

Values of the inhibition constant (*K*_i_) and the putative mechanism of inhibition were derived by comparing the above-mentioned kinetic parameters in the absence or in the presence of fixed concentrations of the extracts, using the following equations for noncompetitive (1) or competitive (2) mechanisms:

*K*_i_ = *V*^′^_max_ × [I]/(*V*_max_ − *V*^′^_max_) (1)

*K*_i_ = *K*_M_ × [I]/(*K*^′^_M_ − *K*_M_) (2)

where *K*^′^_M_ or *V*^′^_max_ represent the *K*_M_ or *V*_max_ measured in the presence of inhibitor concentration [I].

### 2.5. Cell Cultures and Treatments

The human gastric adenocarcinoma AGS and the human neuroblastoma SH-SY5Y cell lines obtained from the American Type Culture Collection (Manassas, VA, USA) were grown in Dulbecco’s modified Eagle medium (DMEM; Microgem Laboratory Research, Milan, Italy), supplemented with 10% heat-inactivated fetal bovine serum (FBS; Microgem Laboratory Research, Milan, Italy), 2 mM L-glutamine, 100 IU/mL penicillin G, and 100 μg/mL streptomycin. The cultures were maintained in a humidified incubator at 37 °C with a 5% CO_2_ environment, plated into 75 cm^2^ dishes every two days, and subjected to the treatments during their exponential growth phase. Cell treatments were performed using non-cytotoxic concentrations [21,31] of *Lo*CT, *Hc*CT, *Ms*F, and *Ci*F (10 or 100 µM) for 24 h after plating.

### 2.6. Total Cell Protein Lysates for Western Blotting Analysis

To prepare the total protein extract, cells were plated in 6-well dishes at a density of 3 × 10^5^ cells per well and incubated for 24 h at 37 °C. Then, cells were treated with two concentrations of each extract (10 or 100 µM) or 0.5% (*v*/*v*) DMSO (Sigma-Aldrich, St. Louis, MO, USA) as a control vehicle. After additional 24 h treatment, cells were collected, rinsed with PBS, and then lysed in an ice-cold modified radioimmunoprecipitation assay (RIPA) buffer (50 mM Tris-HCl, pH 7.4, 150 mM NaCl, 1% Nonidet P-40, 0.25% sodium deoxycholate, 1 mM Na_3_VO_4_, and 1 mM NaF), which was supplemented with protease inhibitors and incubated on ice for 30 min. The supernatant, obtained after centrifugation at 19,100× *g* for 30 min at 4 °C, was used as a total protein extract. The protein concentration was assessed using the Bradford method, with bovine serum albumin (BSA) as a standard. Equal quantities of the total protein extracts (20 µg) were utilized for Western blotting analysis. In brief, the protein samples were mixed with SDS-reducing loading buffer and separated using sodium dodecyl sulfate polyacrylamide gel electrophoresis (SDS–PAGE). Proteins were subsequently transferred to an Immobilon P membrane (Millipore, St. Louis, MO, USA). The membrane was incubated with specific primary antibody at 4 °C overnight, followed by a 1 h incubation at room temperature with the secondary antibody. The primary antibodies used included the human Recombinant Anti-Monoamine Oxidase A/MAO-A antibody [EPR7101] (Abcam, Cambridge, UK), Anti-Monoamine Oxidase B/MAO-B antibody [EPR7102] (Abcam, Cambridge, UK), and the Glyceraldehyde 3-phosphate dehydrogenase antibody (GAPDH) (Cell Signalling Technology, Boston, MA, USA). Membranes were analyzed using an enhanced chemiluminescence reaction with WesternBright ECL (Advansta, San Jose, CA, USA), following the manufacturer’s instructions. Signals were visualized using a Chemidoc MP Imaging System (Bio-Rad, Hercules, CA, USA); densitometric analysis of the signals was assessed using the free image processing software ImageJ, version 1.54D.

### 2.7. Statistical Analysis

Each assay was performed at least three times, and the resulting data were analyzed using the KaleidaGraph program (Synergy, 5.0 version, Adalta, Italy). The data of kinetic and inhibition parameters were reported as the mean ± standard error. Statistical significance of the nonlinear and linear fitting values was calculated with the correlation coefficient R. The statistical significance of data obtained by cell viability was evaluated with the ANOVA, using Bonferroni’s post hoc test; significance was accepted when *p* < 0.05.

## 3. Results

### 3.1. Identification of Organic Compounds Extracted from Leaves of Mediterranean Plants

**Figure 1:**
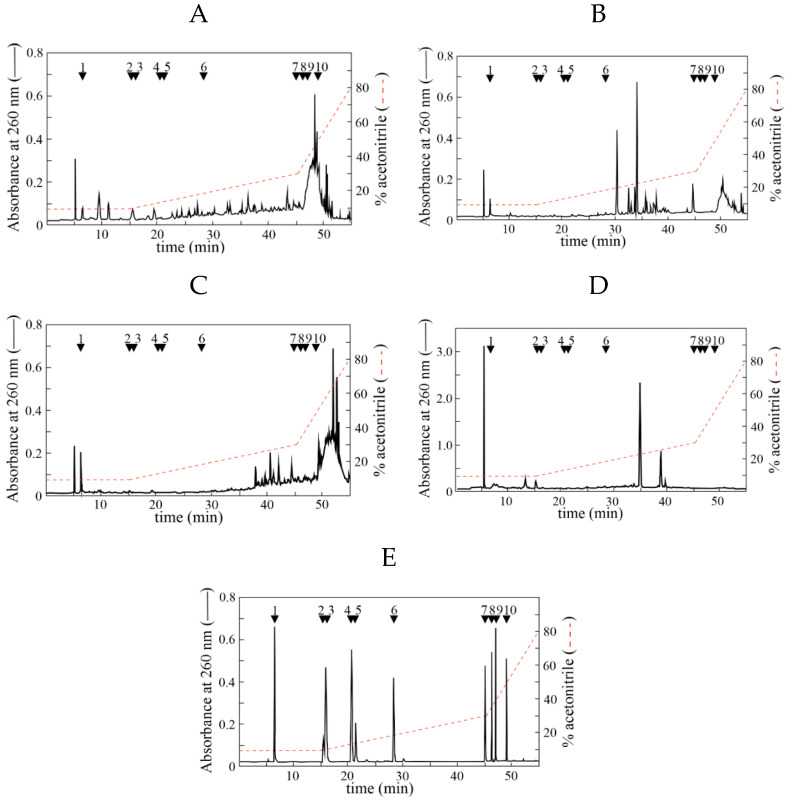
Representative HPLC elution profile of *Lo*CT (**A**), *Hc*CT (**B**), *Ms*F (**C**), *Ci*F (**D**), and standard compounds (**E**). Elution (1 mL/min) was monitored at 260 nm, and the injection volume was 100 µL. The chromatographic run was carried out using the indicated discontinuous gradient and lasted 55 min. Separation of standard compounds (each ~40 nmol) or sample extracts (~50 µg gallic acid equivalent, corresponding to 293 nmol) was achieved in a total run time of 55 min. The list and the corresponding retention times of standard compounds, numbered from 1 to 10, are reported in Table S1. The position of each standard compound is indicated by an arrow.

In a previous work from our group, we have carried out a detailed phytochemical profiling of the extracts from four selected Mediterranean plants, including *Lotus ornithopodioides*, *Hedisarum coronarium*, *Medicago sativa*, and *Cichorium intybus*; indeed, the LC-MS/MS analysis identified and quantified 24 phenolic acids and 25 flavonoids, with a distribution different across the various extracts [21]. To achieve a further focus on the organic compounds contained in these extracts, the samples were also analyzed through an HPLC system. The chromatographic elution profiles of the four extracts and of a mixture of standard compounds are shown in Figure 1. Among the organic compounds identified in the four extracts, *Lo*CT (Figure 1A) and *Ms*F (Figure 1C) had a higher content of hydrophobic components compared to *Hc*CT (Figure 1B) and *Ci*F (Figure 1D). The comparison of these elution profiles with those of standards (Figure 1E) revealed the presence of gallic acid (#1) in all samples, (+)-catechin (#2) in *Lo*CT and *Ci*F, quercetin (#7) in *Hc*CT and *Ms*F, and 7-ethoxycoumarin (#10) in *Lo*CT and *Ms*F. A quantitative analysis indicated that gallic acid (#1) was more abundant in *Ms*F (1.90 ± 0.09 mM) than in *Hc*CT (0.68 ± 0.03 mM), *Lo*CT (0.64 ± 0.05 mM), and *Ci*F (0.10 ± 0.01 mM). Furthermore, the concentration of (+)-catechin (#2), identified only in *Lo*CT and *Ci*F, was 4.80 ± 0.28 and 4.00 ± 0.24 mM, respectively. Finally, quercetin (#7) in *Hc*CT (1.90 ± 0.07 mM) and *Ms*F (1.80 ± 0.11 mM), and 7-ethoxycoumarin (#10) in *Lo*CT (2.80 ± 0.14 mM) and *Ms*F (1.70 ± 0.12 mM) were also quantified. The other standards seem to be absent in the four analyzed extracts. These results are congruent with the composition obtained using the LC-MS/MS analysis previously reported [21].

### 3.2. Effect of Polyphenol-Enriched Plant Extracts on Functionality of Monoamine Oxidases

**Figure 2:**
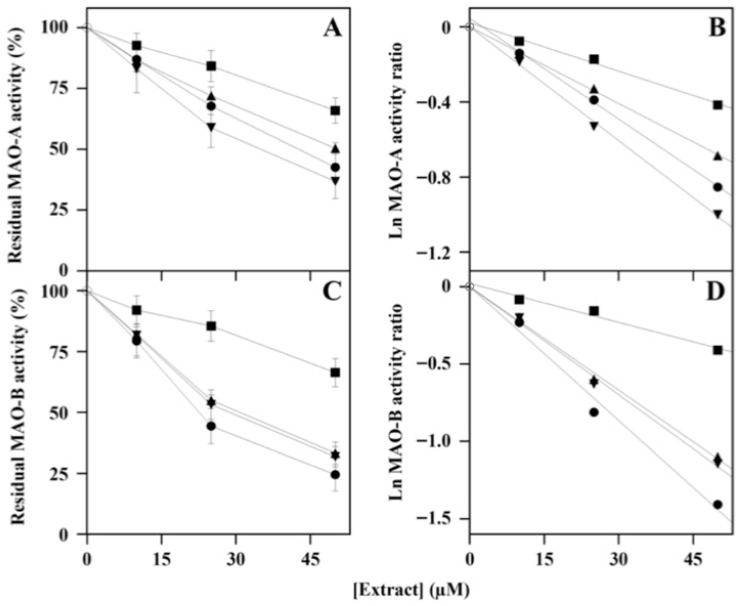
Effect of *Lo*CT, *Hc*CT, *Ms*F, and *Ci*F extracts on the steady-state activity of MAO-A (**A**,**B**) and MAO-B (**C**,**D**). The ratio of activity was measured in the absence (○) or in the presence of the indicated concentrations of *Lo*CT (●), *Hc*CT (▲), *Ms*F (▼), and *Ci*F (■) and expressed as a percentage for MAO-A (**A**) and MAO-B (**C**). The data were also analyzed after a logarithmic transformation of the activity ratio of MAO-A (**B**) and MAO-B (**D**). The correlation coefficient *R* of the linear equation ranged between 0.995 and 0.999 (**B**) or 0.986–0.997 (**D**).

The effects of polyphenols present in the four plant extracts on the steady-state activity of MAOs were evaluated. As shown in Figure 2, all the extracts caused a dose-dependent inhibition of MAO-A and MAO-B, although to a different extent. Regarding the observed effects on MAO-A, no significant differences were found in the inhibition power of the various extracts; *Ms*F and *Ci*F were the most and least effective, respectively (Figure 2A). The logarithmic transformation of the steady-activity data (Figure 2B) allowed a better evaluation of the inhibitory effect through the extrapolation of the IC_50_ values for MAO-A reported in Table 1. In particular, the lowest IC_50_ was assigned to *Ms*F (34 ± 6 µM), closely followed by *Lo*CT (39 ± 8 µM) and *Hc*CT (51 ± 5 µM), and later by *Ci*F (80 ± 18 µM).

Regarding the dose-dependent inhibition profile obtained for MAO-B, all the extracts possessed a slightly greater efficacy compared to MAO-A. Furthermore, the greatest effect was observed with *Lo*CT, whereas *Ci*F remained the least efficient extract (Figure 2C). Also in this case, the different inhibition power was better evaluated with the logarithmic transformation of the activity data (Figure 2D), thus allowing the extrapolation of the corresponding IC_50_ value for MAO-B (Table 1). The data obtained confirm a common greater efficacy of the extracts compared to MAO-A; however, in this case, *Lo*CT was the most powerful in the inhibition (IC_50_, 24 ± 5 µM), followed by *Ms*F (30 ± 3 µM) and *Hc*CT (31 ± 4 µM), and later by *Ci*F (83 ± 19 µM).

**Figure 3:**
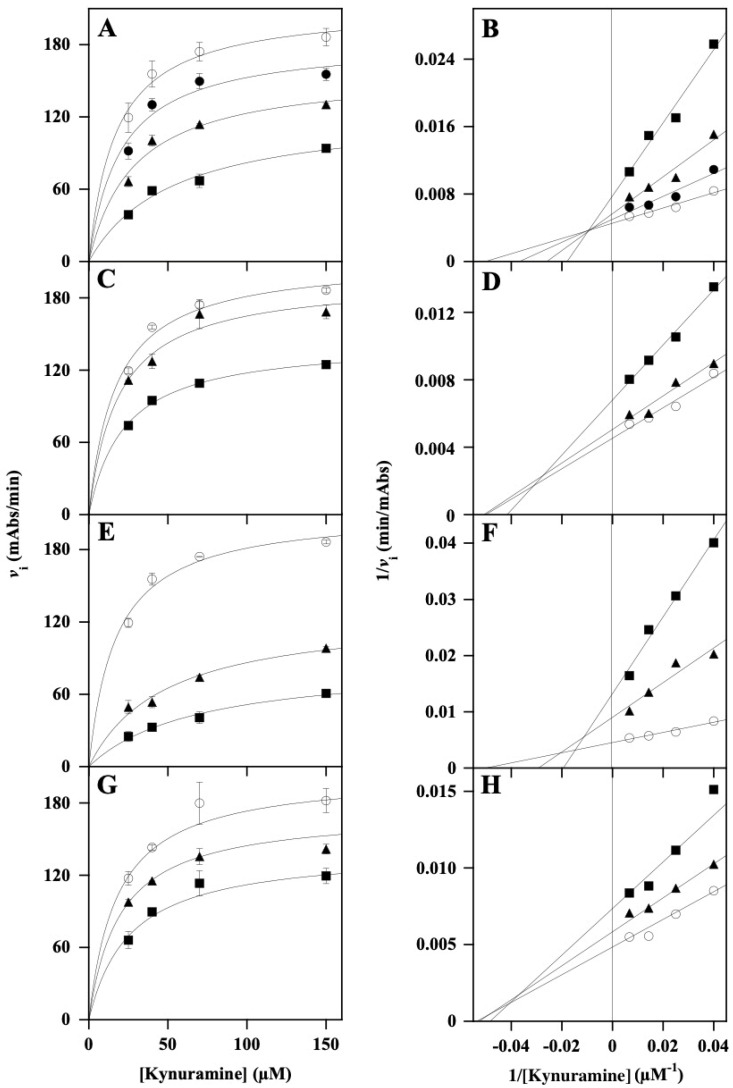
Kinetic analysis of the MAO-A inhibition by *Lo*CT, *Hc*CT, *Ms*F, and *Ci*F extracts. The kinetic measurements of MAO-A activity were realized as reported in the Methods section in the presence of 25–150 µM kynuramine concentration, without (○) or with the following concentration of polyphenol extracts: (**A**,**B**) 10 µM (●), 25 µM (▲), or 50 µM (■) *Lo*CT; (**C**,**D**) 25 µM (▲) or 50 µM (■) *Hc*CT; (**E**,**F**) 25 µM (▲) or 50 µM (■) *Ms*F; and (**G**,**H**) 25 µM (▲) or 50 µM (■) *Ci*F. Data were reported using the hyperbolic Michaelis–Menten equation (**A**,**C**,**E**,**G**) or the Lineweaver–Burk representation (**B**,**D**,**F**,**H**). The correlation coefficient *R* of the hyperbolic or linear equation ranged between 0.948 and 0.985 (**A**,**B**), 0.955–0.996 (**C**,**D**), 0.954–0.992 (**E**,**F**), 0.991–0.999 (**G**,**H**).

The inhibition power and mechanism exerted by the four extracts on MAOs were investigated in more detail through kinetic measurements of MAO-A and MAO-B activity. To this aim, the initial velocity (*v*_i_) of the reaction was measured in the presence of an increasing concentration of the substrate kynuramine, either in the absence or in the presence of fixed concentrations of the various extracts. Kinetic measurements referred to MAO-A are illustrated in Figure 3, where the data were analyzed with the Michaelis–Menten (Figure 3A,C,E,G) and Lineweaver–Burk (Figure 3B,D,F,H) representation. This procedure allowed an evaluation of the effects of the extracts on the *K*_M_ and *V*_max_ of the reaction, whose calculated values were reported in Table 2. Among the four extracts, *Ms*F displayed the greatest effect on the kinetic parameters (Figure 3E), by causing a progressive increase in the *K*_M_ and a concomitant decrease in the *V*_max_, as reported in Table 2. This behavior corresponded to a mixed inhibition mechanism, as also evaluated by the intersection of straight lines in the left negative part of the Lineweaver–Burk plot, above the axis of the substrate (Figure 3F). An evaluation of the inhibition constant (*K*_i_) of *Ms*F towards MAO-A was attempted, and the values obtained through the effects of the extract on *K*_M_ or *V*_max_ were 23.6 ± 2.4 µM or 30.9 ± 1.5 µM, respectively. Although slightly less efficient, the inhibition power of MAO-A by *Lo*CT (Figure 3A) was similar to that exerted by *Ms*F, with a progressive increase in the *K*_M_ and decrease in the *V*_max_, as reported in Table 2. Also, in this case, a mixed inhibition mechanism was suggested, and the calculated *K*_i_ values of *Lo*CT towards MAO-A obtained through the increase in *K*_M_ or decrease in *V*_max_ were 31.7 ± 2.5 µM or 78.9 ± 4.1 µM, respectively (Table 2). These values confirmed a somehow lower efficiency of *Lo*CT compared to *Ms*F; furthermore, positioning of the intersection of the straight lines in the left negative part of the double reciprocal plot, but close to the ordinate axis (Figure 3B), might indicate that *Lo*CT displayed an approaching competitive inhibition mechanism, as also suggested by the lowest value of *K*_i_ obtained with the stronger increase in *K*_M_ compared to the lower decrease in *V*_max_. Concerning the other plant extracts, *Hc*CT (Figure 3C) and *Ci*F (Figure 3G) were much less efficient in the inhibition of MAO-A. Indeed, both *Hc*CT and *Ci*F caused a decrease in the *V*_max_ without a substantial change in the *K*_M_ of the reaction (Table 2), thus suggesting a non-competitive inhibition mechanism, as also indicated by the intersection of straight lines on the abscissa axis for *Hc*CT (Figure 3D) and *Ci*F (Figure 3H). The calculated *K*_i_ values of *Hc*CT (103 ± 2 µM) and *Ci*F (104 ± 5 µM) confirmed the low inhibition power exerted by these extracts on MAO-A.

**Figure 4:**
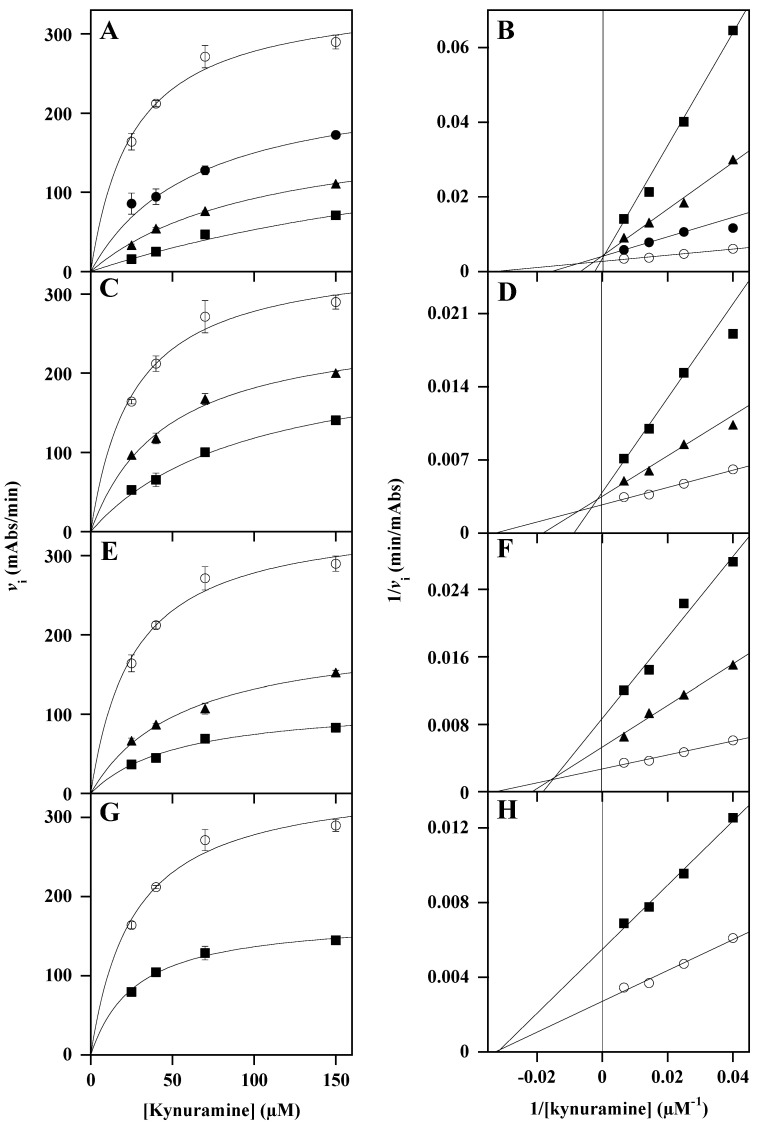
Kinetic analysis of the MAO-B inhibition by *Lo*CT, *Hc*CT, *Ms*F, and *Ci*F extracts. The kinetic measurements of MAO-B activity were realized as reported in the Methods section in the presence of 25–150 µM kynuramine concentration, without (○) or with the following concentration of polyphenol extracts: (**A**,**B**) 10 µM (●), 25 µM (▲), or 50 µM (■) *Lo*CT; (**C**,**D**) 25 µM (▲) or 50 µM (■) *Hc*CT; (**E**,**F**) 25 µM (▲) or 50 µM (■) *Ms*F; (**G**,**H**) 50 µM (■) *Ci*F. Data were reported using the hyperbolic Michaelis–Menten equation (**A**,**C**,**E**,**G**) or the Lineweaver–Burk representation (**B**,**D**,**F**,**H**). The correlation coefficient *R* of the hyperbolic or linear equation ranged between 0.960 and 0.999 (**A**,**B**), 0.980–0.997 (**C**,**D**), 0.980–0.994 (**E**,**F**), and 0.980–0.995 (**G**,**H**).

Moving to the kinetic measurements of MAO-B activity, the corresponding results are illustrated in Figure 4, using the Michaelis–Menten (Figure 4A,C,E,G) or the Lineweaver–Burk (Figure 4B,D,F,H) representation. As a general comment, most of the extracts exerted a greater inhibitory effect on MAO-B compared to MAO-A, and, among them, *Lo*CT (Figure 4A) resulted in the most efficient inhibition power. This extract caused a strong increase in the *K*_M_, leaving unaffected the *V*_max_ of the reaction (Table 3). This behavior clearly indicated that *Lo*CT displayed a competitive inhibition mechanism towards MAO-B, as also suggested by the intersection of straight lines on the ordinate axis of the double reciprocal plot (Figure 4B). The value of *K*_i_ calculated for *Lo*CT (6.4 ± 0.5 µM) confirmed a strong inhibition power possessed by this extract. Although with a lower efficiency, *Hc*CT (Figure 4C) had a behavior like that exerted by *Lo*CT, because it caused a progressive increase in the *K*_M_, leaving unaffected the *V*_max_ of the reaction (Table 3), thus indicating that *Hc*CT also displayed a competitive inhibition mechanism towards MAO-B (Figure 4D). As reported in Table 3, the value of *K*_i_ calculated for *Hc*CT (24.9 ± 2.3 µM) indicates that this extract maintained a good inhibition power towards MAO-B. Concerning *Ms*F (Figure 4E), this extract caused an increase in the *K*_M_ with a concomitant decrease in the *V*_max_ (Table 3), a behavior corresponding to a mixed inhibition mechanism, as also suggested by the double reciprocal plot (Figure 4F). The values of *K*_i_ for *Ms*F calculated through the effects on the *V*_max_ or *K*_M_ were 27.2 ± 1.4 µM or 42.4 ± 4.3 µM, respectively (Table 3), thus indicating that *Ms*F, although using a different mechanism, had an inhibition power similar to *Hc*CT. Interestingly, *Ms*F displayed a similar inhibition power and mechanism on both MAO-A and MAO-B.

Moving to the effects of *Ci*F on MAO-B (Figure 4G), the addition of this extract caused a modest decrease in the *V*_max_ without any change in the *K*_M_ (Table 3), a behavior typical of a non-competitive inhibition mechanism, as also suggested by the intersection of straight lines in the Lineweaver–Burk plot on the abscissa axis (Figure 4H). As reported in Table 3, the calculated *K*_i_ of *Ci*F (48.9 ± 0.6 µM) points to a modest inhibition power on MAO-B, which was, however, greater than that exerted on MAO-A, although with the same inhibition mechanism.

### 3.3. Effect of Polyphenol-Enriched Plant Extracts on MAO-A and MAO-B Protein Expression Level in the Human Gastric Adenocarcinoma AGS and Neuroblastoma SH-SY5Y Cells

**Figure 5:**
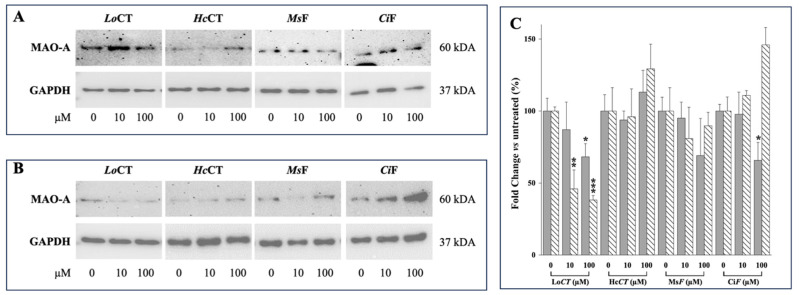
Effect of exposure to *Lo*CT, *Hc*CT, *Ms*F, and *Ci*F extracts on MAO-A protein expression level in AGS and SH-SY5Y cell lines. Cells were treated with the indicated concentration of each extract as reported in the Methods and Materials section. Equal amounts of protein cell lysates (20 μg) were subjected to protein analysis by 12% SDS–PAGE. Western blotting showing MAO-A protein expression levels in AGS (**A**) or SH-SY5Y cells (**B**). GAPDH was used as a loading control for cell lysates. (**C**) Fold change in MAO-A protein levels was calculated by first normalizing to GAPDH levels in individual samples and then relative to untreated control cells cultured in complete DMEM with 0.5% (*v*/*v*) DMSO, as a vehicle, and set to 100 for AGS (gray bars) and SH-SY5Y (striped bars). Each bar represents the mean ± SEM (*n* = 3). Columns with (*, **, ***) were statistically different from untreated control cells (* *p* < 0.05; ** *p* < 0.005; *** *p* < 0.0005, respectively).

Next, we examined whether a treatment with *LoCT*, *HcCT*, *MsF*, and *CiF* extracts could modulate MAO-A and MAO-B protein expression levels in the human gastric adenocarcinoma AGS [32] and neuroblastoma SH-SY5Y [33] cell lines. To this aim, cells were treated with non-cytotoxic concentrations (10 or 100 µM of each extract) for 24 h and then subjected to Western blotting analysis. Our results demonstrated that, among the various extracts, exposure to *Lo*CT induced in both cell lines a great reduction in MAO-A protein expression level compared with that of untreated cells (Figure 5A,B). Densitometric analysis of MAO-A protein expression level (Figure 5C) revealed a significant decrease (20–30%, *p* < 0.05) in AGS cells, and (55–60%, *p* < 0.005 and *p* < 0.0005, respectively) in SH-SY5Y cells, compared to those untreated.

Vice versa, in our experimental conditions, no detectable signals were observed for MAO-B protein expression, either in control or treated AGS and SH-SY5Y cells, although the MAO-B antibody was able to recognize the recombinant MAO-B protein (Supplementary Figure S1).

## 4. Discussion

In recent decades, scientific research efforts have been directed towards the identification of natural substances that can prevent and relieve symptoms of neurodegenerative diseases, such as AD and PD [34,35,36]. Since both disorders are multifactorial, involving different processes and bio-signaling pathways, traditional therapies based on a single drug often fail to counteract the disease progression and induce side effects [37]. In this scenario, a multi-target strategy represents a promising therapeutic perspective, and a great deal has been conducted on plant-derived bioactive compounds or extracts [38,39].

The plants derived extracts contain various phytochemicals, including phenolic compounds such as flavonoids, terpenoids, tannins, and many other molecules; these substances show antioxidant, anti-inflammatory, and anti-amyloidogenic properties, acting in a multitargeting manner as they inhibit both AChE and MAO enzymes [39].

In our previous studies [21,31], based on the same polyphenol-enriched extracts from leaves of *Lotus ornithopodioides* (*Lo*CT), *Hedisarium coronarium* (*Hc*CT), *Medicago sativa* (*Ms*F), and *Chicoriym intybus* (*Ci*F), we reported their composition, rich in condensed tannins or flavonoids, and demonstrated their inhibitory activity on key enzymes involved in the cellular redox balance, such as catalase (CAT) and xanthine oxidase (XO) [31] as well as on acethylcholinesteras (AChE) and butyrilcholinesterase (BuChE) [21]. In the present work, we extended our studies on the inhibitory ability of these extracts on MAO-A and MAO-B. The results pointed to the identification of *Ms*F and *Lo*CT as the best inhibitors of MAO-A (*K*_i_ = 23.6 µM for *Ms*F, and *K*_i_ = 31.7 µM for *Lo*CT), whereas the strongest inhibitor for MAO-B resulted *Lo*CT (*K*_i_ = 6.4 µM). These results are in line with a previous study in which a plant-derived extract inhibited either AChE or MAO-B [40]. These results have been obtained by testing purified MAO enzymes and using polyphenol-enriched extracts, and, therefore, their selectivity toward MAO-A or MAO-B is related to this system model. However, the presence of multiple polyphenol components allows a multitargeting behavior.

On the other hand, we have found that, among the four extracts, *Lo*CT was also able to reduce MAO-A protein expression level in human SH-SY5Y neuroblastoma cells, and, to a lesser extent, in AGS gastric cells.

These findings highlight the potential of natural polyphenol extracts as complementary supplements in the management of neurodegenerative diseases. Notably, the use of antioxidant supplements (e.g., resveratrol, vitamin E, or polyphenol-rich extracts) represents an already widespread practice not only in neurodegeneration but also in cardiovascular, metabolic, and age-related disorders [41]. These supplements highlight the role of oxidative stress in the onset of age-related diseases and the need for managing a healthy lifestyle [42]. Given their multi-targeting effects, not high toxicity, and pleiotropic mechanisms of action, these natural extracts can be considered for further characterization, aiming to develop dietary integration strategies to support conventional therapies.

### Limitations and Future Perspectives

In this study, we demonstrated the inhibitory ability of polyphenol-enriched extracts from Mediterranean plants on MAO-A and MAO-B activity as well as their protein expression level, using in vitro enzyme and cell-based assays; however, some limitations must be acknowledged to contextualize our results and future perspectives. The in vitro enzyme assays and AGS and SH-SY5Y cell lines, although used in studies aiming at the identification of enzyme inhibitors and expression of MAOs-protein, respectively, do not fully mimic the complexity of the human brain and neurodegenerative microenvironment. Similar limitations emerge from other related studies on polyphenol biological properties using in vitro models [43,44] that highlight the importance of more physiologically relevant models; among them, the most used models consist of primary dopaminergic neurons, 3D co-culture models, transgenic animal models of AD and PD, for a better evaluation of neuroprotective efficacy [45].

In addition, the pharmacokinetics and bioavailability of plant polyphenols, particularly their ability to cross the blood–brain barrier [46], remain a critical unresolved issue. In addition, previous studies reported that polyphenols often undergo extensive metabolism, influencing their effects and safety [47,48,49].

In vivo pharmacokinetic assays (e.g., in murine models) will be essential to determine the absorption, distribution, metabolism, and excretion of these compounds. These studies might contribute to assessing potential side effects and interactions with conventional drugs (e.g., synthetic MAO inhibitors) or other enzymatic systems.

Further research could explore the combined effects of these extracts with other neuroprotective compounds (e.g., antioxidants or anti-inflammatory agents) to develop more effective multi-target strategies.

Despite these limitations, our results provide a solid starting point for future studies on multi-target plant-based strategies against MAO-dependent neurodegeneration.

## 5. Conclusions

Our findings demonstrate that polyphenol extracts from Mediterranean plants (*Lo*CT, *Hc*CT, *Ms*F, and *Ci*F) show promising multi-target activity against MAOs, the enzymes involved in neurodegeneration. Among these extracts, *Lo*CT emerges as particularly effective in inhibiting both MAO isoforms and reducing MAO-A protein expression level.

The extracts’ ability to simultaneously modulate MAO activity and expression suggests potential advantages over single-target approaches for complex disorders like AD and PD. The data discussed demonstrate that these extracts possess significant MAO-modulating activity in vitro, positioning them as promising MAO-modulating leads for the isolation of specific compounds and further pharmaceutical investigation. However, the current evidence does not support their instantaneous use in a clinical setting. Rigorous future studies must explicitly address crucial safety and drug–nutrient interaction concerns, including, but not limited to, the potential for interactions with prescribed MAO inhibitors, the risk of inducing serotonin syndrome, or precipitating hypertensive crises, particularly when consumed as part of a tyramine-rich diet. A comprehensive safety profile must be established before any potential clinical applications can be responsibly considered.

While the in vitro results are encouraging, particularly for *Lo*CT, further research should explore their efficacy in animal models and potential clinical translation. This work highlights how Mediterranean plants represent a source of phytochemicals for developing integrative, multi-targeting therapeutic strategies with potentially fewer side effects than synthetic drugs.

## Figures and Tables

**Table 1:** Inhibition by polyphenol extracts on the steady activity of monoamine oxidases.

Extract	MAO-A		MAO-B	
	Concentration Interval (µM)	IC_50_ (µM)	Concentration Interval (µM)	IC_50_ (µM)
*Lo*CT	0–50	39 ± 8	0–50	24 ± 5
*Hc*CT	0–50	51 ± 5	0–50	31 ± 4
*Ms*F	0–50	34 ± 6	0–50	30 ± 3
*Ci*F	0–50	80 ± 18	0–50	83 ± 19

The IC_50_ values were extrapolated from a logarithmic transformation of the activity data.

**Table 2:** Effect of polyphenol extracts on the kinetic parameters of monoamine oxidase A.

Extract	Concentration (µM)	*K*_M_ kynuramine (µM) *	*V*_max_ (mAbs/min) *	Putative Inhibition Mechanism	*K*_i_ (µM)	Calculation of *K*_i_ (From *V*_max_)	*K*_i_ (µM)	Calculation of *K*_i_ (From *K*_M_)
None		18.9 ± 2.1	215 ± 7					
*Lo*CT	10	23.7 ± 3.5	192 ± 8	mixed	31.7 ± 2.5	Equation (1)	78.9 ± 4.1	Equation (2)
	25	33.5 ± 5.1	167 ± 9					
	50	54.3 ± 1.5	127 ± 5					
*Hc*CT	25	19.2 ± 0.5	197 ± 1	non-competitive	103 ± 2			Equation (2)
	50	23.2 ± 0.9	145 ± 2					
*Ms*F	25	41.1 ± 7.0	119 ± 9	mixed	23.6 ± 2.4	Equation (1)	30.9 ± 1.5	Equation (2)
	50	60.2 ± 8.7	81 ± 6					
*Ci*F	25	19.2 ± 0.2	172 ± 1	non-competitive	104 ± 5			Equation (2)
	50	23.2 ± 2.6	138 ± 3					

* Values were obtained by averaging at least three experiments.

**Table 3:** Effect of polyphenol extracts on the kinetic parameters of monoamine oxidase B.

Extract	Concentration (µM)	*K*_M_ kynuramine (µM) *	*V*_max_ (mAbs/min) *	Putative Inhibition Mechanism	*K*_i_ (µM)	Calculation of *K*_i_ (From *V*_max_)	*K*_i_ (µM)	Calculation of Ki (From *K*_M_)
None		28.0 ± 1.7	361 ± 24					
*Lo*CT	10	64.5 ± 0.4	246 ± 1	competitive			6.4 ± 0.5	Equation (2)
	25	127 ± 22	214 ± 24					
	50	380 ± 51	256 ± 29					
*Hc*CT	25	50.6 ± 4.4	275 ± 9	competitive			24.9 ± 2.3	Equation (2)
	50	107 ± 9	245 ± 11					
*Ms*F	25	51.6 ± 5.2	197 ± 9	mixed	42.4.0 ± 4.3	Equation (1)	27.2 ± 1.4	Equation (2)
	50	54.9 ± 0.1	115 ± 1					
*Ci*F	50	29.4 ± 1.8	178 ± 4	non-competitive	48.9 ± 0.6	Equation (1)		

* Values were obtained by averaging at least three experiments.

## Data Availability

The original contributions presented in this study are included in the article/Supplementary Material. Further inquiries can be directed to the corresponding authors.

## Supplementary Materials

The following supporting information can be downloaded at: https://www.mdpi.com/article/10.3390/nu18010022/s1, Figure S1: Western Blotting analysis of MAO-B expression level on total cell protein lysates from AGS and SH-SY5Y cells following the treatment with *Lo*CT, *Hc*CT, *Ms*F and *Ci*F extracts; Table S1: retention times of the standard compounds.
