# Supplementary material for: Polyphenol-Enriched Extracts from Leaves of Mediterranean Plants as Natural Inhibitors of Monoamine Oxidase (MAO)-A and MAO-B Enzymes
Source: Nutrients. 2025 Dec 20;18(1):22. doi: 10.3390/nu18010022 (PMC12787569; doi:10.3390/nu18010022)

Supplementary material

Polyphenol-Enriched Extracts from Leaves of Mediterranean Plants as Natural Inhibitors of Monoamine Oxidase (MAO)-A and MAO-B Enzymes

Antonio D'Errico [1,†], Rosarita Nasso [1,†], Mario Ruggiero [1], Rosario Rullo [2], Emmanuele De Vendittis [3], Mariorosario Masullo [1], Filomena Mazzeo [4,*] and Rosaria Arcone [1,*]

[1] Department of Medical, Movement and Well-Being Sciences, University of Naples "Parthenope", Via Medina, 40, 80133 Napoli, Italy; antonio.derrico002@studenti.uniparthenope.it (A.D.); rosaritanasso@gmail.com (R.N.); mario.ruggiero005@studenti.uniparthenope.it (M.R.); mario.masullo@uniparthenope.it (M.M.)
[2] Institute for the Animal Production Systems in the Mediterranean Environment, Consiglio Nazionale Delle Ricerche, Piazzale Enrico Fermi 1, 80055 Portici, Italy; rosario.rullo@cnr.it
[3] Department of Molecular Medicine and Medical Biotechnologies, University of Naples Federico II, Via S. Pansini 5, 80131 Napoli, Italy
[4] Department of Economics, Law, Cybersecurity and Sport Sciences, University of Naples "Parthenope", Via Della Repubblica, 32, 80035 Nola, Italy
* Correspondence: filomena.mazzeo@uniparthenope.it (F.M.); rosaria.arcone@uniparthenope.it (R.A.)
† These authors contributed equally to this work.

Table S1 – Retention times of the standard compounds

Figure S1 – Western Blotting analysis of MAO-B expression level on total cell protein lysates from AGS and SH-SY5Y cells following the treatment with *Lo*CT, *Hc*CT, *Ms*F and *C*iF extracts.

Table S1. Retention times of the standard compounds used in the HPLC analysis

#	Standard compound	Retention time (min)
1	gallic acid	6.32
2	(+)-catechin	15.29
3	chlorogenic acid	15.78
4	caffeic acid	20.50
5	(-)-epicatechin	21.25
6	coumaric acid	28.33
7	quercetin	45.15
8	(E)-cinnamic acid	46.36
9	2-fluorocinnamic acid	47.00
10	7-ethoxycoumarin	49.09

Figure S1. Western Blotting analysis of MAO-B expression level in AGS and SH-SY5Y cells following the treatment with LoCT, HcCT, MsF and CiF extracts. On the SDS-PAGE were loaded gel 20 μg of total cell protein lysates from the treated cells and 0.25 μg of MAO-B (see Material and Methods sections 2.1. and 2.5.).

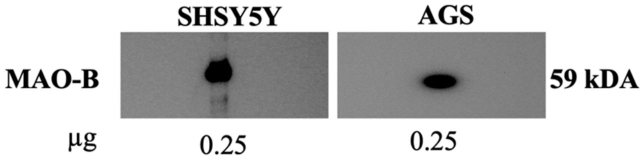

Supplement: Supplementary file 1 [file nutrients-18-00022-s001.zip › nutrients-4030257-supplementary.pdf]
